# Predicting Cognitive Decline in Amyloid-Positive Patients With Mild Cognitive Impairment or Mild Dementia

**DOI:** 10.1212/WNL.0000000000209605

**Published:** 2024-07-10

**Authors:** Pieter J. van der Veere, Jeroen Hoogland, Leonie N.C. Visser, Argonde C. Van Harten, Hanneke F. Rhodius-Meester, Sietske A.M. Sikkes, Vikram Venkatraghavan, Frederik Barkhof, Charlotte E. Teunissen, Elsmarieke van de Giessen, for the Alzheimer's Disease Neuroimaging Initiative (ADNI),, Johannes Berkhof, Wiesje M. Van Der Flier

**Affiliations:** From the Alzheimer Center and Department of Neurology (P.J.v.d.V., L.N.C.V., A.C.V.H., H.F.R.-M., S.A.M.S., V.V., W.M.V.D.F.), and Department of Epidemiology and Biostatistics (P.J.v.d.V., J.H., L.N.C.V., J.B., W.M.V.D.F.), Amsterdam Neuroscience, VU University Medical Center; Amsterdam Neuroscience (P.J.v.d.V., L.N.C.V., A.C.V.H., H.F.R.-M., V.V., C.E.T., E.G., W.M.V.D.F.), Neurodegeneration the Netherlands; Division of Clinical Geriatrics (L.N.C.V.), Center for Alzheimer Research, Department of Neurobiology, Care Sciences and Society, Karolinska Institutet, Stockholm, Sweden; Medical Psychology (L.N.C.V.), Amsterdam UMC Location AMC, University of Amsterdam; Amsterdam Public Health (L.N.C.V.), Quality of Care, Personalized Medicine; Internal Medicine (H.F.R.-M.), Geriatric Medicine Section, Amsterdam Cardiovascular Sciences Institute, Amsterdam UMC Location VUmc; Department of Clinical, Neuro and Developmental Psychology (S.A.M.S.), Faculty of Movement and Behavioral Sciences, VU University; Department of Radiology & Nuclear Medicine (F.B., E.G.), Amsterdam UMC, Vrije Universiteit, the Netherlands; Queen Square Institute of Neurology and Centre for Medical Image Computing (F.B.), University College London, United Kingdom; and Neurochemistry Laboratory and Biobank (C.E.T.), Department of Clinical Chemistry, Amsterdam Neuroscience, VU University Medical Center, the Netherlands.

## Abstract

**Background and Objectives:**

Cognitive decline rates in Alzheimer disease (AD) vary greatly. Disease-modifying treatments may alter cognitive decline trajectories, rendering their prediction increasingly relevant. We aimed to construct clinically applicable prediction models of cognitive decline in amyloid-positive patients with mild cognitive impairment (MCI) or mild dementia.

**Methods:**

From the Amsterdam Dementia Cohort, we selected amyloid-positive participants with MCI or mild dementia and at least 2 longitudinal Mini-Mental State Examination (MMSE) measurements. Amyloid positivity was based on CSF AD biomarker concentrations or amyloid PET. We used linear mixed modeling to predict MMSE over time, describing trajectories using a cubic time curve and interactions between linear time and the baseline predictors age, sex, baseline MMSE, *APOE* ε4 dose, CSF β-amyloid (Aβ) 1–42 and pTau, and MRI total brain and hippocampal volume. Backward selection was used to reduce model complexity. These models can predict MMSE over follow-up or the time to an MMSE value. MCI and mild dementia were modeled separately. Internal 5-fold cross-validation was performed to calculate the explained variance (*R*^2^).

**Results:**

In total, 961 participants were included (age 65 ± 7 years, 49% female), 310 had MCI (MMSE 26 ± 2) and 651 had mild dementia (MMSE 22 ± 4), with 4 ± 2 measurements over 2 (interquartile range 1–4) years. Cognitive decline rates increased over time for both MCI and mild dementia (model comparisons linear vs squared vs cubic time fit; *p* < 0.05 favoring a cubic fit). For MCI, backward selection retained age, sex, and CSF Aβ1–42 and pTau concentrations as time-varying effects altering the MMSE trajectory. For mild dementia, retained time-varying effects were Aβ1–42, age, *APOE* ε4, and baseline MMSE. *R*^2^ was 0.15 for the MCI model and 0.26 for mild dementia in internal cross-validation. A hypothetical patient with MCI, baseline MMSE 28, and CSF Aβ1–42 of 925 pg/mL was predicted to reach an MMSE of 20 after 6.0 years (95% CI 5.4–6.7) and after 8.6 years with a hypothetical treatment reducing decline by 30%.

**Discussion:**

We constructed models for MCI and mild dementia that predict MMSE over time. These models could inform patients about their potential cognitive trajectory and the remaining uncertainty and aid in conversations about individualized potential treatment effects.

## Introduction

Alzheimer disease (AD) is a progressive neurodegenerative disease with considerable variability in the rate of cognitive decline.^1^ The disease is highly prevalent, with roughly 100 million people estimated to be in the mild cognitive impairment (MCI) and dementia stages of the disease.^2^ From the MCI stage, it is estimated to take 4 years on average before people have progressed to dementia.^3^ New disease-modifying treatments targeting amyloid plaques slow disease progression in the MCI and mild dementia stages of AD.^4-6^ However, the clinical meaningfulness of these medications is debated.^7^ Two factors in this debate are the challenge of translating the identified 30% reduction in decline rates into outcomes relevant to patients and the complexity of assessing the impact of disease-modifying treatments on an individual's decline trajectory because of heterogeneity in progression.

Patients are highly interested in their expected disease course.^8,9^ To accommodate these needs, prediction models of individualized natural cognitive trajectories and the associated uncertainty are urgently needed. When these individualized natural course predictions are combined with intervention efficacy data, the putative intervention benefits can be personalized.

Predicting progression from MCI to dementia has received much attention in the literature.^10^ While the future risk of dementia can be predicted with reasonable precision using MRI and CSF biomarker information,^10-12^ this crude end point may not be the most meaningful to patients. In addition, patients with mild dementia do not benefit from these predictions while prognostic information is equally important to them. In a study on outcomes that matter to patients with AD and their caregivers, participants indicated cognitive decline to be among the most important factors.^8^ Earlier models predicting cognitive decline have been published.^12-14^ However, they are either limited to patients with MCI^12^ or the models have not been built for easy clinical use.^13,14^ Therefore, we aimed to construct clinically applicable prediction models of cognitive decline in amyloid-positive patients with MCI or mild dementia.

## Methods

### Design and Patients

In this longitudinal study, we included participants from the Amsterdam Dementia Cohort, which is a mixed memory clinic cohort of all patients with memory complaints presenting at Alzheimer Center Amsterdam. While the Amsterdam Dementia Cohort does not have exclusion criteria, elderly patients are often referred to the geriatric outpatient clinic and thus do not present themselves for inclusion in the cohort. Inclusion criteria for this study were a baseline diagnosis of MCI or mild dementia (clinical dementia rating of less than 2), amyloid positivity at baseline, and a baseline and at least 1 follow-up Mini-Mental State Examination (MMSE). Participants had their baseline visit between August 2002 and December 2022. This study followed the Transparent Report of a Multivariable Prediction Model for Individual Prognosis or Diagnosis reporting guideline.

At our memory clinic, a standardized 1-day diagnostic workup is performed, including medical history; neurologic, physical, and neuropsychological tests; MRI; and lumbar puncture.^15^ This includes measurements of height, weight, systolic and diastolic blood pressure, and information on depression with the Geriatric Depression Scale,^16^ education on the Verhage scale,^17^ and smoking history. Diagnosis of dementia due to AD and MCI was made in a multidisciplinary meeting.^18^ All diagnoses fulfilled the core clinical criteria, National Institute on Aging-Alzheimer's Association criteria.^19,20^ During annual follow-up, medical examination and neuropsychological tests were performed without blinding to information gathered at baseline.

We used the MMSE as the main cognitive outcome in this study.^21^ Those with MCI had a median follow-up of 3 (interquartile range [IQR] 2–5) years with on average 4 (SD 2) MMSE measurements per participant for a combined 1,315 measurements. The mild dementia group had a median of 2 (IQR 1–3) years of follow-up with an average of 3 (SD 2) MMSE measurements per participant for a combined 2,113 measurements. As an additional outcome to provide information on the decline in memory, we used the Dutch version of the Rey Auditory Verbal Learning Test (RAVLT) Immediate Recall score (total available: n = 2,855, MCI: n = 1,227, mild dementia: n = 1,628).^22^

### Amyloid Positivity and CSF Measurements

Amyloid positivity was defined based on either AD biomarkers in CSF or on amyloid PET within 6 months after the baseline MMSE. β-Amyloid (Aβ) 1–42 and phosphorylated threonine 181 (pTau) information from CSF was available in 874 (91%) participants. Before 2018, sandwich ELISA was used (Innotest, Fujirebio, Gent, Belgium). Innotest Aβ values were drift corrected.^23^ From 2018 onward, CSF was analyzed using Elecsys (Roche, Rotkreuz, Switzerland). For the Innotest assays, a drift-corrected Aβ1–42 below 813 pg/mL was considered positive, and for Elecsys assays, a pTau/Aβ1–42 ratio of more than or equal to 0.020 was considered positive.^24^ For the prediction models, Innotest CSF values were bridged to Elecsys.^25^ In total, 860 participants were amyloid positive based on their CSF.

Amyloid PET imaging was performed for 309 (32%) participants using 3-Tesla Ingenuity TF PET/MRI, Ingenuity TF PET/CT, and Gemini TF PET/CT scanners (Philips Healthcare, Amsterdam, the Netherlands) with the 11C-Pittsburgh compound B, 18F-flutemetamol, and 18F-florbetaben compounds.^26,27^ Visual rating was performed according to company guidelines or for 11C-Pittsburgh compound B according to previously published methods and discriminated between positive scans (n = 297) and negative scans.^28^

Two hundred twenty-two (23%) participants had both CSF and PET measurements. Participants could be included when either their CSF or amyloid PET was positive. CSF and PET were concordant for 196 (20%) participants and discordant for 26 (3%) based on positive CSF and negative amyloid PET (n = 12) or vice versa (n = 14).

### MRI Measurements

MRI was performed on site in 762 (79%) participants. Before 2008, 1-T and 1.5-T scanners were used (Magnetom Avanto, Impact, and Sonata, Siemens; Signa, GE Healthcare). From 2008 onward, 3-T scanners were used (Magnetom Siemens; Discovery MR750, Signa GE Medical Systems; Ingenuity TF PET/MR, Philips Medical Systems; and Titan, Toshiba Medical Systems). All scans were performed using a standardized protocol.^29^

Volumetric MRI measurements were the primary MRI biomarkers used in this study. Left and right hippocampal volume and whole brain volume were quantified using Freesurfer version 7.1 (available in 709 [74%] participants), visually checked, and scanner-related differences were adjusted for thorough harmonization using the ComBat procedure.^30^

### Statistical Analyses

Baseline information was missing for some participants (eTable 1) on all predictors except for age, sex, and baseline MMSE and diagnosis. Missing information was imputed using multiple imputations by chained equations in 25 imputation data sets. Variables used in the identification of donors were selected based on a minimal correlation of 0.05 with the variable being imputed. Baseline diagnosis was used in all donor selections. Parameter estimates were pooled across the imputation sets. The distribution of imputed values and convergence were assessed visually.

We used linear mixed models to model MMSE over time, including a random slope and intercept per individual. Separate models were developed for MCI and mild dementia. First, the trajectory of MMSE over time (including baseline in the outcome) was described using only a cubic time curve. Subsequently, we used backward selection procedures to construct models predicting MMSE over follow-up using a cubic time curve and baseline measurements. The baseline measures could be included as predictors with a constant effect over time (no interaction with time) or as predictors with a time-varying effect (interaction of linear time × baseline predictor). In these models, baseline MMSE was included as a potential predictor. Backward selection was started from (1) a base model including age, sex, and baseline MMSE; (2) a biomarker model adding to the base model: CSF Aβ1–42 and pTau, MRI total brain and hippocampal volume, and *APOE* ε4; or (3) a full model adding a range of clinical variables and risk factors encompassing the Verhage score, Geriatric Depression Scale, systolic and diastolic blood pressure, body mass index (categorized as <25, 25–30, and >30 kg/m^2^), and smoking history. The various models represent variations in information availability in clinical settings. Variables that were selected (*p* < 0.10) in at least half of the 25 imputed sets were included in the final models pooled over all imputed sets. The time, age, and sex variables were preselected in all imputation sets.

We investigated the effect of the statistical method used by evaluating 2 additional modeling approaches without backward selection: no parameter penalization and ridge penalization. Both statistical methods were applied to the 3 models listed above. For ridge penalization,^31^ local shrinkage was applied in 4 groups: the cubic time curve, MMSE at baseline, other parameters without time-varying effects, and parameters with linear time-varying effects.

Predictive performance was assessed using internal 5-fold cross-validation for the no parameter penalization, backward selection, and ridge penalization models. Out-of-sample root mean squared error, median absolute deviation, and proportion of explained variance (*R*^2^) were calculated. Internal cross-validation of ridge penalization was performed using 5 imputed data sets because of computational limitations. For MCI and mild dementia, 1 statistical method and model (base, biomarker, full model) was selected to highlight based on the model performance and the number of parameters included in the models, favoring a more parsimonious model.

We visualized the predicted decline pattern in the MCI and mild dementia groups in one of the imputed data sets (arbitrarily the first) based on the estimated fixed and random effects, highlighting the 2nd, 16th, 50th, 84th, and 98th percentiles of predicted decline at different time points. To visualize the interindividual variation (error) and provide insight into the uncertainty surrounding individualized predictions, we plotted 1,000 samples from the random effect distribution around the predicted mean MMSE for a hypothetical patient with MCI and mild dementia. The hypothetical patients were based on the median predictor values in each group. The decline is also shown with hypothetical interventions that reduce the predicted mean MMSE decline by 10%, 30%, and 50%.

To simplify the interpretation of the predicted decline, we used the model to estimate the time to reach a threshold MMSE of 20 (indicating mild dementia) for MCI and 15 for moderate dementia.^32^ The time to the threshold MMSE was calculated for different baseline CSF Aβ1–42 and baseline MMSE measurements; other predictors were fixed at the median. In addition, we provide the time to threshold MMSE with a hypothetical intervention that reduced decline by 30%.

We performed external validation for all constructed models in data from Alzheimer's Disease Neuroimaging Initiative (ADNI).^33^ ADNI is a longitudinal self-referral scientific cohort of patients with cognitively unimpairment, MCI, and dementia aged 55–99 years.^33^ Important exclusion criteria were the presence of significant neurologic disease other than AD or prior psychiatric diagnoses interfering with the cognitive assessments. The cohort was launched in 2003 to test whether serial MRI, PET, other biological markers, and clinical and neuropsychological assessment can be combined to measure the progression of MCI and early AD. Baseline and follow-up measurements in ADNI included all information needed to validate the models. No visual MRI read information was available from ADNI. From ADNI, participants who met the inclusion criteria were included for this study (see eTable 2 for baseline characteristics). For the MCI sample, we selected those with “late” MCI.^34^ In total, 598 ADNI participants were included (389 MCI; 209 mild dementia), with 2 (IQR 1–4) years of follow-up and 4 (IQR 3–6) MMSE measurements on average. The mean age was 74 years (SD 8), and 41% were female.

The modeling and validation steps were also performed with RAVLT as the outcome (see eMethods for additional information). Exploratory analyses using visual MRI read biomarkers and excluding CSF as potential predictors are included in eMethods and eResults.

The highlighted models are available as a shiny app as a proof of concept for the implementation of prediction tools on *predictmmse.com*. Normally distributed variables are displayed with means and SD and skewed distributions with medians and IQRs. Model diagnostics were assessed graphically. All analyses were performed in R version 4.2.1,^35^ with the use of the “lme4,” “mgcv,” and “mice” packages.

### Standard Protocol Approvals, Registrations, and Patient Consents

The study protocol of the Amsterdam Dementia Cohort was approved by the ethical review board of the VU University Medical Center (2016.061). Written informed consent was obtained from all patients for the use of their data for research purposes.

### Data Availability

Data can be made available upon reasonable request.

## Results

Within the Amsterdam Dementia Cohort, there were 1,789 amyloid-positive participants with MCI (n = 436) or mild dementia (n = 1,344) and a baseline MMSE measurement. Of those, 961 participants also had a follow-up MMSE measurement (Table 1), 310 of whom had MCI and 651 had mild dementia; 462 (48%) were female; over 90% were White, with an average age (SD) of 65 years (7). The mild dementia group without follow-up had a 1.7 (SE 0.2) point lower baseline MMSE and 93 (SE 12) pg/mL lower CSF Aβ1–42 concentration (eTable 3) than the group with follow-up.

**Table 1:** Baseline Characteristics

	Total (n = 961)	Partition based on diagnosis	
		MCI (n = 310)	Mild dementia (n = 651)
Age at baseline, y, mean ± SD	65 ± 7	66 ± 7	65 ± 7
Female, n (%)	461 (48.0)	141 (45.5)	320 (49.2)
MMSE at baseline, mean ± SD	23.6 ± 3.9	26.5 ± 2.3	22.3 ± 3.8
No. of MMSE measurements, mean ± SD	3.6 ± 1.8	4.2 ± 2.1	3.2 ± 1.5
Years between first and last MMSE, median (IQR)	2.2 (1.2–3.6)	3.1 (2.0–4.9)	2.0 (1.1–3.1)
RAVLT Immediate Recall at baseline, mean ± SD	25.3 ± 8.5	30.2 ± 7.3	22.9 ± 8.1
Years of education,^a^ mean ± SD	12.2 ± 3.0	12.5 ± 3.1	12.0 ± 2.9
Geriatric Depression Scale, median ± IQR	2.8 ± 2.4	3.0 ± 2.5	2.7 ± 2.3
Systolic blood pressure, mm Hg, mean ± SD	147 ± 19	146 ± 19	148 ± 19
Diastolic blood pressure, mm Hg, mean ± SD	84 ± 10	84 ± 10	84 ± 10
Body mass index, kg/m^2^, n (%)			
Below 18.5	18 (2.0)	3 (1.0)	15 (2.5)
18.5–25	522 (57.6)	157 (53.2)	365 (59.7)
25–30	303 (33.4)	118 (40.0)	185 (30.3)
Over 30	63 (7.0)	17 (5.8)	46 (7.5)
Reported smoking status, n (%)			
Never	473 (49.7)	146 (47.6)	327 (50.8)
Stopped	331 (34.8)	108 (35.2)	223 (34.6)
Current smoker	147 (15.5)	53 (17.3)	94 (14.6)
CSF measures			
β-Amyloid 1–42, pg/mL, mean ± SD	757 ± 210	789 ± 217	742 ± 205
Phosphorylated tau, pg/mL, median (IQR)	32.2 (23.8–43.2)	29.7 (22.9–41.0)	33.5 (24.3–45.3)
MRI measures			
Medial temporal atrophy score, mean ± SD	1.1 ± 0.8	0.8 ± 0.7	1.3 ± 0.8
Global atrophy score, mean ± SD	0.9 ± 0.6	0.7 ± 0.6	1.0 ± 0.7
Fazekas score, mean ± SD	1.0 ± 0.8	1.0 ± 0.7	1.0 ± 0.8
Lacunes visually present, n (%)	152 (20.7)	63 (25.0)	89 (18.4)
Microbleeds visually present, n (%)	62 (8.2)	27 (10.5)	35 (7.0)
Total brain volume, mL, mean ± SD	1,078 ± 110	1,102 ± 113	1,066 ± 106
Hippocampal volume, mL, mean ± SD	6.8 ± 0.9	7.0 ± 0.9	6.6 ± 0.9
*APOE* ε4 alleles, n (%)			
0	247 (26.4)	67 (22.4)	180 (28.3)
1	447 (47.8)	140 (46.8)	307 (48.3)
2	241 (25.8)	92 (30.8)	149 (23.4)

Abbreviations: IQR = interquartile range; MMSE = Mini-Mental State Examination; RAVLT = Rey Auditory Verbal Learning Test.

Baseline characteristics shown before imputation.

a Years of education were calculated from the Verhage score.

In both MCI and mild dementia, the yearly decline in MMSE increased during follow-up (model comparisons for linear vs squared, and squared vs cubic time fit; *p* < 0.05). In MCI, the average MMSE declined from 26.4 (95% CI 26.2–26.7) to 25.8 (25.5–26.1) after 18 months, to 24.2 (23.7–24.6) after 3 years, and to 21.0 (20.2–21.7) after 5 years. In mild dementia, the average MMSE declined from 22.4 (95% CI 22.0–22.7) to 19.8 (19.4–20.2), 15.3 (14.7–15.9), and 7.8 (6.8–8.9), respectively.

Internal cross-validation indicated that the models captured some of the variation in decline in MMSE in patients with MCI and mild dementia, albeit considerable uncertainty remained (Table 2). The backward selected models performed comparably with the models without penalization and had slightly lower performance than the ridge penalization model (eTables 4–6), but we highlight the backward selected models because they use fewer parameters. In the backward selected models, the biomarker model (model 2) performed slightly better than the basic model (model 1) and similar to the full model (model 3), albeit with fewer parameters. Thus, we focus on the biomarker model in the following sections. In the MCI group, the mean out-of-sample *R*^2^ and median absolute deviation in internal validation (Table 2) were 0.17 and 2.05, respectively, and in the mild dementia group, 0.26 and 2.83. This means that in half of the predictions made for patients with MCI, the observed MMSE deviated by less than 2 points from the predicted MMSE. Correspondingly, the deviation was less than approximately 3 points in mild dementia.

**Table 2:** Predictive Performance of the MMSE Prediction Models in Internal Cross-Validation

Model	No. of parameters (n; range over folds)	RMSE (range over folds)	MAD (range over folds)	*R*^2^ (range over folds)
Mild cognitive impairment				
Base model				
No penalization	10	3.67 (3.36–3.97)	2.17 (1.84–2.75)	0.06 (−0.15 to 0.24)
Backward selection	8 (7–8)	3.62 (3.38–3.96)	2.13 (1.85–2.50)	0.09 (−0.15 to 0.23)
Ridge	10	3.52 (3.20–3.93)	2.09 (1.84–2.59)	0.15 (−0.09 to 0.26)
Biomarker model				
No penalization	18	3.48 (3.15–3.88)	2.06 (1.77–2.50)	0.16 (−0.04 to 0.30)
Backward selection	13 (11–15)	3.47 (3.13–3.85)	2.05 (1.71–2.58)	0.17 (−0.08 to 0.28)
Ridge	18	3.39 (3.12–3.84)	2.07 (1.84–2.54)	0.21 (0.01 to 0.32)
Full model				
No penalization	36	3.49 (3.12–3.88)	2.14 (1.78–2.46)	0.16 (−0.02 to 0.32)
Backward selection	15 (13–19)	3.51 (3.17–3.88)	2.11 (1.86–2.58)	0.14 (−0.10 to 0.30)
Ridge	36	3.38 (3.08–3.86)	2.07 (1.76–2.51)	0.22 (0.02 to 0.34)
Mild dementia				
Base model				
No penalization	10	4.77 (4.20–5.25)	2.90 (2.80–3.01)	0.22 (0.05 to 0.42)
Backward selection	9 (8–9)	4.75 (4.19–5.24)	2.90 (2.80–3.10)	0.22 (0.05 to 0.41)
Ridge	10	4.48 (4.06–4.87)	2.78 (2.73–2.87)	0.31 (0.18 to 0.47)
Biomarker model				
No penalization	20	4.65 (4.14–5.16)	2.83 (2.56–3.02)	0.26 (0.08 to 0.42)
Backward selection	13 (12–15)	4.65 (4.12–5.14)	2.83 (2.55–2.96)	0.26 (0.09 to 0.42)
Ridge	20	4.44 (4.03–4.92)	2.75 (2.62–2.86)	0.32 (0.17 to 0.48)
Full model				
No penalization	36	4.68 (4.17–5.14)	2.78 (2.46–3.03)	0.25 (0.09 to 0.44)
Backward selection	19 (17–22)	4.69 (4.20–5.14)	2.84 (2.56–2.99)	0.24 (0.09 to 0.43)
Ridge	36	4.40 (3.97–4.86)	2.70 (2.46–2.86)	0.33 (0.18 to 0.50)

Abbreviations: Aβ = β-amyloid; MAD = median absolute deviation; RMSE = root mean squared error.

The different prediction models were 5-fold internal cross-validation using all imputed data sets. Basic predictors included time, time^2^, time^3^, MMSE at baseline, age, sex, and the interaction of all these variables with a linear fit of time. Biomarker predictors also included *APOE* ε4 allele count, CSF Aβ1–42 and pTau, MRI normalized total brain and hippocampal volume, and the interaction of all these variables with a linear fit of time. The full model also included Geriatric Depression Scale, Verhage score, smoking history, body mass index, systolic and diastolic blood pressure, and their interactions with a linear fit of time. For the ridge prediction models, normalization of the variables was performed based on the mean and standard deviation of the variables in the imputed Amsterdam Dementia Cohort. For the ridge cross-validation, only 5 imputed data sets were used.

Different variables were retained in the MCI and mild dementia groups (Table 3, eTables 4, and 6). In MCI, baseline MMSE was retained as a time-constant effect and retained time-varying effects were age, sex, and CSF pTau and Aβ1–42. In mild dementia, retained time-constant effects were sex and CSF pTau and retained time-varying effects were age, baseline MMSE, *APOE* ε4, and CSF Aβ1–42. Volumetric MRI information was retained in none of the biomarker models.

**Table 3:** Regression Coefficients of the Backward Selected Prediction Models of MMSE Over Time in MCI and Mild Dementia

Variable names	MCI	Mild dementia
	Coefficient (SE)	Coefficient (SE)
Intercept	12.9446 (2.4238)	−10.2450 (2.4825)
Years since baseline^a^	−1.6700 (1.1730)	−9.9597 (1.4078)
Years since baseline squared^a^	−0.2090 (0.0486)	−0.2196 (0.0582)
Years since baseline cubed^a^	0.0141 (0.0091)	0.0227 (0.0139)
Age at baseline, y	0.0147 (0.0239)	0.1020 (0.0274)
Sex, reference female	0.4329 (0.3227)	−0.0811 (0.2718)
MMSE at baseline	0.5283 (0.0534)	1.0006 (0.0561)
No. of *APOE* ε4 alleles	—	0.5419 (0.2842)
CSF pTau, pg/mL log transformed	−1.1162 (0.4153)	−1.0403 (0.2990)
CSF Aβ1–42, pg/mL	0.0008 (0.0008)	0.0019 (0.0010)
Interaction of years since baseline		
Age at baseline, y	0.0188 (0.0142)	0.0376 (0.0156)
MMSE at baseline	—	0.1299 (0.0345)
No. of *APOE* ε4 alleles	—	0.4768 (0.1636)
CSF Aβ1–42, pg/mL	0.0006 (0.0005)	0.0009 (0.0006)
Sex, reference female	0.2884 (0.1926)	—
CSF pTau, pg/mL log transformed	−0.3894 (0.2484)	—

Abbreviations: Aβ = β-amyloid; MCI = mild cognitive impairment; MMSE = Mini-Mental State Examination.

In backward selection, the cubic time curve, age, and sex were forced into the model. The other variables were backward selected based on a *p*-value of <0.10 and were included in the final model if the variables were selected in at least half the imputed data set. Parameter estimates and standard error are based on pooling between the imputed data sets.

a Centered by subtracting 2.3.

**Figure 1:**
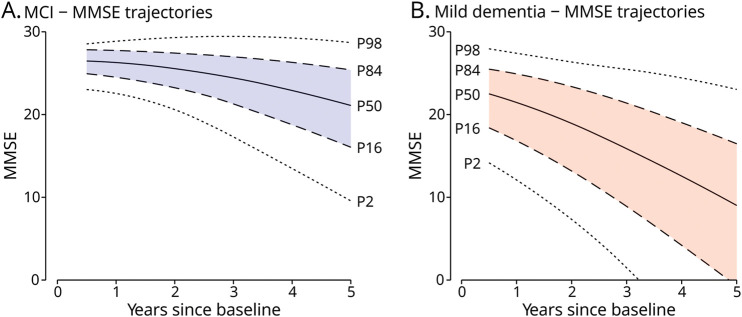
Distribution of Predicted MMSE Trajectories in the MCI and Mild Dementia Groups MMSE trajectories over time were estimated for all participants based on their patient characteristic and estimated random intercept and slope using the backward selected prediction models for mild cognitive impairment (A) and mild dementia (B) using the biomarker model. Arbitrarily, complete data from the first imputed data set were used. From all estimated MMSE trajectories, the predicted MMSE measurements at the 98th, 84th, 50th, 16th, and 2nd percentiles are plotted. Predicted MMSE measurements outside of the possible range (0–30) have not been plotted. Predicted MMSE is first displayed after 6 months. MCI = mild cognitive impairment, MMSE = Mini-Mental State Examination.

We visualized the heterogeneity in predicted decline in the study cohort (Figure 1) to allow patients to compare themselves with a representative “population.” The 84th (+1 SD from the mean) and 16th (−1 SD from the mean) percentiles of the predicted mean MMSE in the MCI group after 9 months were 28.0 and 24.7, respectively. After 5 years, the predicted mean MMSE measurements were 25.4 (84th percentile) and 16.2 (16th percentile). The 84th and 16th percentiles of the predicted mean MMSE in the mild dementia group after 9 months were 25.3 and 17.5, respectively, and 21.5 and 8.4 after 3 years. Against this population distribution, individualized predictions can be shown.

**Figure 2:**
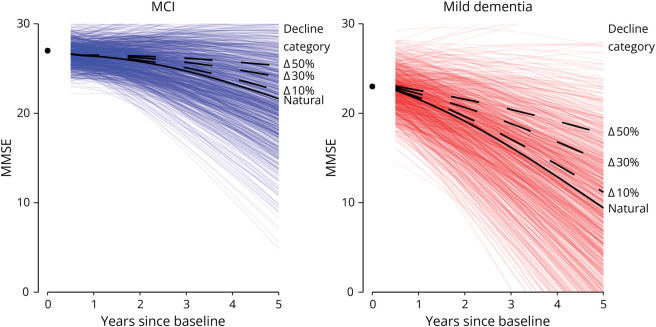
Simulated Interindividual Variation Surrounding Predicted MMSE Trajectories Depicts predicted MMSE trajectories for hypothetical patients with MCI and mild dementia with median predictor values in each group. For the MCI group, the fixed parameter values were sex: male; age: 66 years; log CSF pTau: 3.415 ng/L; CSF Aβ1–42: 796.22; and baseline MMSE 27. For the mild dementia group, the fixed parameters values were sex: female; age: 66 years; *APOE* ε4 alleles: 2; log CSF pTau: 3.498 ng/L; CSF Aβ1–42: 758.8; and baseline MMSE 23. Predictions were made using the model based on the backward selection biomarker model. The black solid line is the predicted mean MMSE trajectory based on the median values, the “natural” trajectory. The colored lines surrounding the predicted MMSE depict 1,000 simulated random intercepts and effects, indicating interindividual variation in MMSE trajectories not explained by the available predictors. The dashed lines show expected MMSE trajectories with interventions that reduce decline by 10%, 30%, or 50%, respectively. The circle at year 0 indicated the baseline MMSE. Predicted MMSE is first presented after 6 months of follow-up. Aβ = β-amyloid; MCI = mild cognitive impairment; MMSE = Mini-Mental State Examination.

Next, we visualized the estimated trajectory with the unexplained interindividual variation (Figure 2). For the hypothetical patient with MCI with median predictor values, the predicted mean MMSE after 5 years was 21.0 (95% CI 20.9–23.0). By drawing 1,000 samples from the random-effect distribution, we can visualize the unexplained interindividual variation surrounding this predicted MMSE, showing 90% was between 30 and 13 MMSE points, indicating substantial variation for individuals with the same predictor values. As a next step, the MMSE predictions can also be made in the hypothetical situation where an intervention reduces decline by 30%. For this patient with MCI, a 30% reduction in decline would give a predicted mean MMSE after 5 years of 23.7. Within the interindividual variation surrounding the natural decline, this reduced decline places at the 69th percentile of the distribution. So, compared with the natural decline trajectory of 100 patients with MCI with the same predictor values, 69 are likely to have a lower MMSE after 5 years and 31 a higher MMSE. With a steeper predicted natural decline, the projected effect of interventions that reduce decline deviates more from the unexplained interindividual variation in MMSE (Figure 2).

**Figure 3:**
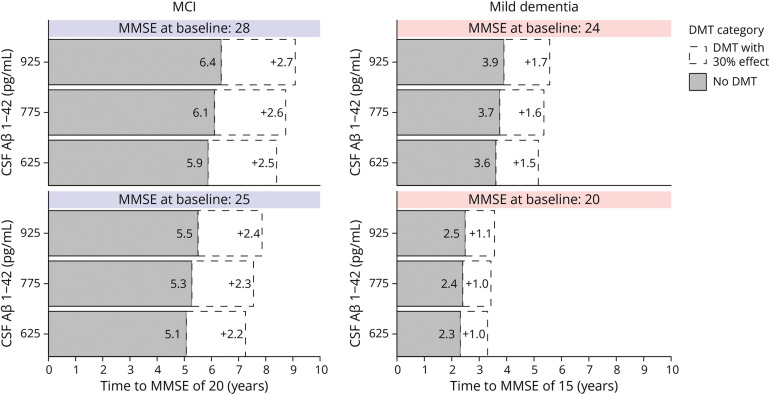
Time to a Further Cognitive Stage Time to reach a threshold MMSE of 20 and 15 was calculated using the backward selected biomarker model. The median value of all selected predictors in the MCI and mild dementia groups was used, varying only CSF Aβ1–42 and MMSE at baseline. The CSF Aβ1–42 values approximately reflect the P25, P50, and P75 values in the overall cohort. For the MCI group, the fixed parameters values were sex: male; age: 66 years; and log CSF pTau: 3.415 ng/L. For the mild dementia group, the fixed parameter values were sex: female; age: 66 years; *APOE* ε4 alleles: 2; and log CSF pTau: 3.498 ng/L. The white areas indicate the expected additional time to reach the threshold MMSE with a hypothetical DMT that reduces decline by 30%. The statistical uncertainty surrounding the time estimates is left out for visual clarity and is given in the text. Aβ = β-amyloid; DMT = disease-modifying treatment; MCI = mild cognitive impairment; MMSE = Mini-Mental State Examination.

To make the results of the models more intuitive, we also visualized the models as a personalized predicted time to a certain MMSE value (Figure 3). The predicted mean time to reach an MMSE of 20 for a patient with MCI with a baseline MMSE of 28 and CSF Aβ1–42 of 925 pg/mL was 6.0 years (95% CI 5.4–6.7 years). For a patient with mild dementia with a baseline MMSE of 20 and CSF Aβ1–42 of 625 pg/mL, the predicted mean time to reach an MMSE of 15 was 2.3 years (95% CI 2.1–2.5). These estimates can also be used to evaluate potential time gains with inventions that reduce the rate of decline. With a hypothetical intervention that reduces decline by 30%, the time to threshold would be 8.6 years for the patient with MCI and 3.3 years for the patient with mild dementia.

External validation of all prediction models in ADNI showed comparable performance between the model-building approaches (eTable 7). The mild dementia backward selected biomarker model (model 2) had an *R*^2^ of 0.20 and median absolute deviation of 2.19 in ADNI. The MCI model had an *R*^2^ of 0.21 and median absolute deviation of 1.97 (eTable 7).

In an additional set of analyses, we constructed prediction models for RAVLT Immediate Recall (eTables 8–10). Compared with the backward selected MMSE models, fewer variables were retained with time-varying effects. In internal cross-validation, RAVLT models in participants with MCI performed comparably with MMSE models with *R*^2^ ranging between 0.11 and 0.20 (eTable 11). Performance of the RAVLT models in mild dementia was slightly better than that of the MMSE models, with an *R*^2^ ranging between 0.32 and 0.34. This is also reflected in the external validation of the RAVLT models in ADNI, where the *R*^2^ of the mild dementia models clustered around 0.50 (eTable 12). The *R*^2^ of the MCI RAVLT models in ADNI ranged between 0.25 and 0.33.

## Discussion

We constructed clinically applicable prediction models of cognitive decline measured by MMSE or RAVLT for patients with amyloid-positive MCI and mild dementia. Adding MRI and CSF biomarkers to base variables somewhat improved predictions, although the modest explained variance illustrates that making individualized predictions inherently comes with uncertainty. Our models can be used to predict the time to reach a certain level of MMSE or RAVLT. We incorporate these models in a calculator with visualization as a prototype tool to discuss prognosis, the uncertainty surrounding the predictions, and the impact of intervention strategies with patients.

The overall predictive performance of the models for both MCI and mild dementia indicates a substantial amount of variation in MMSE decline could already be explained by clinical variables age, sex, baseline MMSE, and time since baseline. Additional information on MRI volumetric and CSF Aβ1–42 and pTau biomarkers, representing etiologic disease characteristics,^36^ aided in the prediction of MMSE decline in our amyloid-positive sample. However, further increasing model complexity by adding other clinical and vascular risk factors did not improve predictive performance despite their known association with AD dementia.^37,38^ Potentially, tau PET information could improve predictive performance because of the association with AD-associated symptom severity,^39^ but we could not incorporate this because of a lack of data.

Compared with other studies that predicted MMSE decline, our models showed similar^12^ or even better^13,14^ predictive performance while requiring less^12^ or similar^13,14^ information. Two studies used MCI Biofinder patients^13^ or amyloid-positive ADNI patients^12^ to build prediction models for MMSE decline, after 2 and 4 years, based on demographic and plasma biomarker information^13^ or a wide variety of CSF, MRI volumetric, cognitive test, and vascular risk factor information.^12^ The “AD course map” model used cognitively normal patients or those with MCI clinical AD from ADNI and jointly modeled decline in cognition, PET hypometabolism and MRI cognitive thinning, and hippocampal deformation.^14^ One study predicted decline in functional impairment through the Clinical Dementia Rating Sum of Boxed Score and showed similarly modest predictive performance compared with the various MMSE studies.^40^ These former studies used different statistical techniques, namely linear regression,^12,13^ multivariate nonlinear mixed-effect models,^14^ gradient boosting,^13,40^ and different ways to weigh data.^13^ We investigated localized shrinkage with ridge regression and no penalization of linear mixed model coefficients as additional approaches, which both did not substantially alter the predictive performance compared with backward selection. Our finding that the statistical approach did not have a strong effect on the predictive performance follows the relative equivalence in performance from different statistical models in the literature. This implies 2 things. First, additional predictors are needed to capture the remaining unexplained variation. A possible avenue would be combining clinical predictors and polygenetic risk scores.^41^ Second, the use of novel statistical techniques does not result in large gain in performance. Thus, keeping models simple and the way in which the model can be used is more relevant than the small performance variation between techniques. The linear mixed model approach we used allows for prediction at any point in time within the data range (approximately 5 years in this study) and visualization of different sources of uncertainty in individualized prediction.

Patients with AD and their care partners want to know their future cognitive functioning.^8^ Our prediction models can be used to inform patients about their cognitive decline, but our results also indicate that providing a precise prognosis is challenging. Thus clinicians need to talk about the inherent uncertainty surrounding the predictions with their patients.^42^ Visualizations of the uncertainty can form the basis for meaningful doctor-patient conversations about the predicted cognitive decline.^43^

In the communication of prognostic information to patients, a link needs to be made between the answers models can provide and the questions patients and their care partners have such as “how long can I still drive a car” or “how long can I in my hobby.”^8^ The MMSE provides an indication of global cognition and does not answer these questions. However, no currently available cognitive test addresses all the questions patients have or takes into consideration the diversity in patients' living situations affecting the extent to which they can use their remaining cognitive function.^44^ In the future, we hope prediction models will become available directly predicting patient-reported outcomes such as quality of life and daily functioning. Such data are currently being collected,^45^ but long-term follow-up is needed to develop robust models. Until then, there is an important role for clinicians in translating the observed and predicted cognitive function scores into answers to patients' questions. We attempted to aid clinicians by translating the rate of decline into a clinically meaningful outcome by providing estimations of time to a certain MMSE level.

In both the analysis of the interindividual variation in decline and time to a certain MMSE level, we added hypothetical medication effects. By calculating the “additional” time to a certain MMSE level when slowing decline with hypothetical interventions, we provide an easier way to think about clinical meaningfulness than absolute changes in memory score. At the same time, these figures visualize that benefits be difficult to distinguish from variation in natural decline patterns. The applied hypothetical interventions extrapolate beyond the time frame of the amyloid-targeting therapy trial results.^4-6^ We assumed the effects would be stable over time and across disease stages or patient subgroups such as APOE subtype or sex. These assumptions could be inappropriate and long-term follow-up of patients undergoing treatment is essential to further refine such models in the future.

Strengths of this study include, first, the large, real-world population used to build the prediction models. We selected our sample to include patients who in theory could be eligible for the novel generation of disease-modifying treatments, i.e. patients with amyloid-positive MCI and mild dementia. In addition, participants were included from a tertiary memory clinic, a setting in which these interventions are likely to be implemented first. This makes our study highly relevant in helping shape the future patient journey.^46^ Second, we used straightforward statistical methods, improving the interpretability and acceptability of the final biomarker prediction models in a clinical setting. For clinical applicability, parsimonious and simple models are preferred over more complex statistical models.

There are some limitations that warrant discussion. First, we used MMSE as a measure of cognition because of the short time it takes to collect in the clinic and its widespread use. However, MMSE measurements show intraindividual variation in a cognitively normal population.^47^ Furthermore, MMSE measurements in our clinic are not always taken at the same time of day and patients with cognitive decline might score lower later in the day when they are more tired.^48^ Both factors increase unexplainable noise in the outcome, reducing predictability. As an alternative, we also modeled RAVLT as a second outcome. Contrary to what might have been expected, we did not find higher predictive accuracy for RALVT than for MMSE. Second, the models were built for use in memory clinics based on tertiary memory clinic data. Thus, generalizability to the general population could be limited. Although external validation in ADNI did not show diminished performance, indicating generalizability to an older population, MCI or dementia due to AD in the general population is likely to occur in individuals with more comorbidities than are present in either the development or validation cohort. Third, the selected mild dementia population had a slightly higher baseline MMSE score and Aβ1–42 than the average patient with amyloid-positive mild dementia in our cohort. However, as the rate of MMSE decline in mild dementia is modulated by these 2 predictors, the generalizability of the predictions should not be affected. Fourth, no information was available on the number of impaired cognitive domains from participants with MCI. This information may have improved predictions.^49^

We constructed clinically applicable models to predict MMSE and memory over time in patients with MCI or mild dementia due to AD. There is a need among patients and care partners for prognostic information on their cognitive trajectory. These models can provide such information, although our results also emphasize that the heterogeneity in cognitive trajectories can only be partially captured. The models come with an easy-to-use calculator allowing visualization of the predicted cognitive trajectories. Such a tool can form the basis for a meaningful discussion about patients' expected natural decline trajectory and how initiating hypothetical intervention strategies might alter this decline.

## Appendix Authors

**Table:**

Name	Location	Contribution
Pieter J. van der Veere, MD	Alzheimer Center and Department of Neurology, Department of Epidemiology and Biostatistics, Amsterdam Neuroscience, VU University Medical Center; and Amsterdam Neuroscience, Neurodegeneration, the Netherlands	Drafting/revision of the manuscript for content, including medical writing for content; study concept or design; analysis or interpretation of data
Jeroen Hoogland, MD	Department of Epidemiology and Biostatistics, Amsterdam Neuroscience, VU University Medical Center, the Netherlands	Drafting/revision of the manuscript for content, including medical writing for content; analysis or interpretation of data
Leonie N.C. Visser, PhD	Alzheimer Center and Department of Neurology, Department of Epidemiology and Biostatistics, Amsterdam Neuroscience, VU University Medical Center; and Amsterdam Neuroscience, Neurodegeneration, the Netherlands; Division of Clinical Geriatrics, Center for Alzheimer Research, Department of Neurobiology, Care Sciences and Society, Karolinska Institutet, Stockholm, Sweden; Medical Psychology, Amsterdam UMC Location AMC, University of Amsterdam; Amsterdam Public Health, Quality of Care, Personalized Medicine, the Netherlands	Drafting/revision of the manuscript for content, including medical writing for content
Argonde C. Van Harten, MD, PhD	Alzheimer Center and Department of Neurology, Amsterdam Neuroscience, VU University Medical Center; Amsterdam Neuroscience, Neurodegeneration, the Netherlands	Drafting/revision of the manuscript for content, including medical writing for content
Hanneke F. Rhodius-Meester, MD, PhD	Alzheimer Center and Department of Neurology, Amsterdam Neuroscience, VU University Medical Center; Amsterdam Neuroscience, Neurodegeneration; Internal Medicine, Geriatric Medicine Section, Amsterdam Cardiovascular Sciences Institute, Amsterdam UMC Location VUmc, the Netherlands	Drafting/revision of the manuscript for content, including medical writing for content
Sietske A.M. Sikkes, PhD	Alzheimer Center and Department of Neurology, Amsterdam Neuroscience, VU University Medical Center; Department of Clinical, Neuro and Developmental Psychology, Faculty of Movement and Behavioral Sciences, VU University, the Netherlands	Drafting/revision of the manuscript for content, including medical writing for content
Vikram Venkatraghavan, PhD	Alzheimer Center and Department of Neurology, Amsterdam Neuroscience, VU University Medical Center; Amsterdam Neuroscience, Neurodegeneration, the Netherlands	Drafting/revision of the manuscript for content, including medical writing for content; analysis or interpretation of data
Frederik Barkhof, MD, PhD, FRCR	Department of Radiology & Nuclear Medicine, Amsterdam UMC, Vrije Universiteit, the Netherlands; Queen Square Institute of Neurology and Centre for Medical Image Computing, University College London, London, United Kingdom	Drafting/revision of the manuscript for content, including medical writing for content; major role in the acquisition of data
Charlotte E. Teunissen, PhD	Amsterdam Neuroscience, Neurodegeneration; Neurochemistry Laboratory and Biobank, Department of Clinical Chemistry, Amsterdam Neuroscience, VU University Medical Center, the Netherlands	Drafting/revision of the manuscript for content, including medical writing for content; major role in the acquisition of data
Elsmarieke van de Giessen, MD, PhD	Amsterdam Neuroscience, Neurodegeneration; Department of Radiology & Nuclear Medicine, Amsterdam UMC, Vrije Universiteit, the Netherlands	Drafting/revision of the manuscript for content, including medical writing for content; major role in the acquisition of data
Johannes Berkhof, PhD	Department of Epidemiology and Biostatistics, Amsterdam Neuroscience, VU University Medical Center, the Netherlands	Drafting/revision of the manuscript for content, including medical writing for content; study concept or design; analysis or interpretation of data
Wiesje M. Van Der Flier, PhD	Alzheimer Center and Department of Neurology, Department of Epidemiology and Biostatistics, Amsterdam Neuroscience, VU University Medical Center; and Amsterdam Neuroscience, Neurodegeneration, the Netherlands	Drafting/revision of the manuscript for content, including medical writing for content; major role in the acquisition of data; study concept or design; analysis or interpretation of data

## Glossary

Aβ: β-amyloid
AD: Alzheimer disease
ADNI: Alzheimer's Disease Neuroimaging Initiative
IQR: interquartile range
MCI: mild cognitive impairment
MMSE: Mini-Mental State Examination
RAVLT: Rey Auditory Verbal Learning Test
